# Endothelial Differentiation of Human Adipose-Derived Stem Cells on Polyglycolic Acid/Polylactic Acid Mesh

**DOI:** 10.1155/2015/350718

**Published:** 2015-05-28

**Authors:** Meng Deng, Yunpeng Gu, Zhenjun Liu, Yue Qi, Gui E. Ma, Ning Kang

**Affiliations:** ^1^Liposuction Center of Plastic Surgery Hospital, Chinese Academy of Medical Sciences and Peking Union Medical College, Beijing 100144, China; ^2^Research Center of Plastic Surgery Hospital, Chinese Academy of Medical Sciences and Peking Union Medical College, Beijing 100144, China

## Abstract

Adipose-derived stem cell (ADSC) is considered as a cell source potentially useful for angiogenesis in tissue engineering and regenerative medicine. This study investigated the growth and endothelial differentiation of human ADSCs on polyglycolic acid/polylactic acid (PGA/PLA) mesh compared to 2D plastic. Cell adhesion, viability, and distribution of hADSCs on PGA/PLA mesh were observed by CM-Dil labeling, live/dead staining, and SEM examination while endothelial differentiation was evaluated by flow cytometry, Ac-LDL/UEA-1 uptake assay, immunofluorescence stainings, and gene expression analysis of endothelial related markers. Results showed hADSCs gained a mature endothelial phenotype with a positive ratio of 21.4 ± 3.7% for CD31+/CD34− when induced in 3D mesh after 21 days, which was further verified by the expressions of a comprehensive range of endothelial related markers, whereas hADSCs in 2D induced and 2D/3D noninduced groups all failed to differentiate into endothelial cells. Moreover, compared to 2D groups, the expression for *α*-SMA was markedly suppressed in 3D cultured hADSCs. This study first demonstrated the endothelial differentiation of hADSCs on the PGA/PLA mesh and pointed out the synergistic effect of PGA/PLA 3D culture and growth factors on the acquisition of mature characteristic endothelial phenotype. We believed this study would be the initial step towards the generation of prevascularized tissue engineered constructs.

## 1. Introduction

In regenerative medicine and tissue engineering, vascularization plays an important role in tissue repair and it is also critical to the survival of newly regenerated tissues such as bone, adipose, and skin [1–6]. Besides common surgical techniques, conventional strategies to promote engineered constructs vascularization are as follows: the applications of growth factors or angiogenesis-inducing biomaterials [7, 8] to recruit endothelial progenitor cells and the direct employment of endothelial cells to establish a coculture system [9]. However, due to the short half-life of growth factors and the limited acquisition of endothelial cells, investigators have to seek alternative approaches to incorporate vasculature within engineered constructs.

Adipose-derived stem cell (ADSC) has shown great prospects in cell-based tissue regeneration due to its abundance and easy accessibility following minimally invasive procedures. ADSC possesses the capacity similar to bone marrow mesenchymal stem cell (BMSC) to differentiate into osteogenic, adipogenic, myogenic, and chondrogenic lineages under appropriate conditions [10]. Besides, Bekhite et al. [11] addressed that ADSC could gain an endothelial phenotype when cultured on a matrigel surface under hypoxia, VEGF, and leptin. Additionally, ADSCs were able to express a comprehensive range of specific endothelial markers under certain shear stress [12, 13]. Recently, an angiogenic therapy based on ADSC in an ischemic model has also been reported [14]. These evidences suggested the important advantages of ADSC for cell-based therapy in restoring endothelial functions and promoting vascularization.

Polyglycolic acid (PGA) is FDA approved and successfully used to construct tissues such as cartilage, bone, tendon, muscle, and skin [15–17] in tissue engineering. However, the unwoven fiber is incapable of maintaining a fixed configuration and it is too soft to bear pressure load. In our study, PGA fibers were coated with polylactic acid (PLA) in order to maintain a fixed porous structure and gain a better stiffness [18]. As these two biomaterials were both given approval by FDA, the PGA/PLA complex might be a promising candidate with a broad applicability as a cell scaffold or delivery vehicle in the regeneration of various tissues [15, 19].

To the best of our knowledge, there were no reports on the endothelial differentiation of ADSCs seeded on PGA/PLA mesh. Considering the angiogenic potential of ADSCs, the combined application of ADSCs and PGA/PLA compound will provide a general tissue construction model featuring the angiogenic effects, which might be of great help to the repair of ischemic tissue or the vasculature integration of newly regenerated tissues to the host site. Thus in the present study, we systematically investigated the endothelial differentiation of human ADSCs on 3D PGA/PLA mesh compared to 2D plastic under a supplement of VEGF by flow cytometry, Ac-LDL/UEA-1 uptake assay, immunofluorescence stainings of vWF, VEGFR2, and CD31, and gene expression analysis of endothelial related markers. Besides, the cytoskeleton related marker of *α*-SMA was examined to further understand the effect of PGA/PLA 3D culture on the endothelial differentiation. We believe this would be the initial step towards the generation of prevascularized tissue engineered constructs.

## 2. Materials and Methods

### 2.1. Isolation and Culture of Human ADSCs (hADSCs)

Human adipose tissue was obtained from liposuction procedures (*n* = 4) in compliance with the guidelines of Ethics Committee of Plastic Surgery Hospital. The lipoaspirates were washed twice, digested with 0.1% (w/v) type I collagenase (Sigma, USA) with agitation at 37°C for 30 minutes, passed through a 74 *μ*m filter, and finally selected on the basis of adherence to plastic culture flasks. Cells were grown in MSC culture medium (Sciencell, USA) supplemented with 5% fetal bovine serum, 1% MSC growth supplement, 100 U/mL penicillin, and 100 g/mL streptomycin in a 5% CO_2_ incubator at 37°C, and medium was changed every 3 days. HADSCs cultured at passage 3 were used for subsequent experiments.

### 2.2. Fabrication of PGA/PLA Scaffold

10 milligrams of PGA unwoven fibers (provided by National Tissue Engineering Research Center, Shanghai, China) was compressed into a cylinder shape with 5 mm diameter and 0.8 mm thickness (*n* = 30). A solution of 1% (w/v) PLA (Sigma, USA) diluted in dichloromethane was evenly dropped onto the PGA fibers to solidify each scaffold until the PLA reached 20% weight of per PGA mesh. The scaffolds were sterilized by 75% alcohol and washed 3 times with PBS followed by DMEM with 10% FBS overnight.

### 2.3. Flow Cytometry Analysis

The surface markers of P_3_ hADSCs were characterized by flow cytometry (BD FACS Aria, Germany) with specific fluorescein isothiocyanate (FITC), phycoerythrin (PE), Peridinin-chlorophyll-protein complex (PerCP), or allophycocyanin- (APC-) conjugated monoclonal antibodies, including CD90, CD105, CD73, CD44, CD29, CD166, CD106, CD45, CD34, CD31, SSEA1, and SSEA4 (BD Biosciences, USA). To evaluate the endothelial phenotype of ADSC cultured on PGA/PLA, the 3D complexes after 7, 14, and 21 days of induction were harvested and digested with 0.25% trypsin for 5 min followed by 0.1% type I collagenase (Sigma, USA) for 15 min to release the cells from polymer, and then the 2D/3D induced cells were tested by mouse anti-human CD34-APC (eBioscience, USA) and mouse anti-human CD31-PE (eBioscience, USA). The protocols were as follows: cells were resuspended with 100 *μ*L buffer (PBS containing 0.1% bovine serum albumin) and incubated for 30 min on ice with the above antibodies. After washing with PBS, the cells were resuspended in the acquisition PBS containing 1% formaldehyde until analysis. In each run, at least 10,000 events were acquired with the FACS Arial II flow cytometer (BD, USA) and the results were analyzed by Flowjo software. HUVECs (purchased from Sciencell, USA) served as the positive control.

### 2.4. SEM Examination

The PGA/PLA mesh and hADSCs-PGA/PLA complexes after 14 days of in vitro culture were subjected to SEM examination. Each of the complexes was fixed with 2.5% cool glutaraldehyde for 30 min, air-dried overnight, and gold-sputter-coated for observation.

### 2.5. Biomechanical Evaluation

A biomechanical analyzer (Tytron 250, MTS, USA) was used for mechanical tests. Samples of PGA/PLA mesh and PGA fiber (*n* = 3/group) were compressed into a cylinder shape and measured with diameter and thickness. A constant compressive strain rate of 0.05 mm/s was applied until 30% of maximal deformation was achieved and a stress-strain curve was generated. Young's modulus of the tested samples was calculated based on the formula, *Y* = *S* × *T*/*A* (*Y*: Young's modulus, *S*: slope of stress-strain curve, *T*: thickness, and *A*: superficial area).

### 2.6. CM-Dil Labeling and Live/Dead Staining

P_3_ hADSCs were labeled by being immersed into 10 mM CM-Dil (Invitrogen, UK) for 5 min at 37°C followed by 15 min at 4°C and observed at 3 days after seeding by optical microscopy. The cell viability on PGA/PLA mesh was detected using the live/dead cell staining kit (BioVision, USA) after 21 days of induction by confocal laser scanning microscopy (Zeiss, Germany).

### 2.7. In Vitro Culture and Endothelial Differentiation of hADSC

P_3_ hADSCs were seeded onto plastic dish at 2.5 × 10^4^/cm^2^ or resuspended with a density of 10 × 10^6^/mL and seeded onto PGA/PLA scaffolds, respectively. They were cultured either in completed medium (DMEM supplemented with 10% FBS, 100 U/mL penicillin, and 100 g/mL streptomycin) or in endothelial differentiation medium (EGM-2-MV, LONZA, USA) supplemented with 20 ng/mL VEGF (Peprotech, USA) for 21 days in vitro. Medium in these 4 groups was all changed every 3 days.

### 2.8. Acetylated-Low-Density Lipoprotein/Ulex europaeus-1 (Ac-LDL/UEA-1) Uptake

After 21 days of induction, LDL uptake was detected by incubating the 2D and 3D cultured samples in medium for 4 h at 37°C with 2 mg/mL of Ac-LDL labeled with Dil (Invitrogen, USA). Then, they were washed, fixed in 4% polyoxymethylene, and incubated with FITC-conjugated UEA-1 (Sigma Aldrich, USA) 100 mg/mL for 1 h at 37°C. Finally, 2D and 3D induced samples were visualized by confocal laser scanning microscopy, respectively. Noninduced 2D and 3D cultured cells incubated in completed medium (DMEM supplemented with 10% FBS, 100 U/mL penicillin, and 100 g/mL streptomycin) served as the negative controls while HUVEC served as the positive control.

### 2.9. Immunofluorescence Stainings

After 21 days of induction, the 2D and 3D cultured samples were rinsed with PBS and subjected to immunofluorescence stainings. After fixing with ice-cold methanol for 5 min, the sections were permeabilized with 0.1% Triton-X100 for 5 min, blocked with 1% BSA for 10 min, and then incubated with rabbit polyclonal anti-vWF antibody (1 : 100, Abcam, UK), rabbit polyclonal anti-VEGFR2 antibody (1 : 100, Novus, USA), and rabbit monoclonal anti-CD31 antibody (1 : 100, Novus, USA) for 2 h at 37°C. After 3 washes in PBS, Texas Red goat anti-rabbit IgG (1 : 300, Molecular Probes, USA) was added for 1 h. Complexes were incubated with DAPI for nuclei staining. Noninduced 2D and 3D hADSCs incubated in completed medium (DMEM supplemented with 10% FBS, 100 U/mL penicillin, and 100 g/mL streptomycin) served as the negative controls while HUVEC served as the positive control. Besides, the immunofluorescence staining of *α*-SMA (1 : 100, Bioss, China) was examined in these 4 groups after 7 days in vitro. All of the samples were examined under optical microscopy.

### 2.10. Gene Expression Analysis

The 2D and 3D cultured ADSCs with or without induction in the 4 groups were individually collected at 7, 14, and 21 days (*n* = 3 per group) and the total RNA was extracted using trizol reagent (Invitrogen, USA), respectively. RNA was reverse transcribed into single-stranded cDNA according to the manufacturer's protocol (Promega, USA). The gene expression levels of vWF, VEGF, and eNOS were analyzed by quantitative PCR using a LightCycler 480 system with a SYBR green kit (Roche Molecular Biochemicals, Germany). Also, the mRNA expression levels of *α*-SMA in these 4 groups after 7 days in vitro were detected. The forward and reverse primer pairs were shown in Table 1. The housekeeping gene of GAPDH was amplified to be as an internal control.

### 2.11. Statistical Analysis

The data were analyzed using Student's *t*-test (for Young's modulus) and one-way ANOVA test (for gene expression) by SPSS Statistics 17.0 software. Data were presented as mean ± SEM (standard error of the mean), and a *P* value less than 0.05 was considered statistically significant.

## 3. Results

### 3.1. Characterization of hADSCs by Flow Cytometry

**Figure 1:**
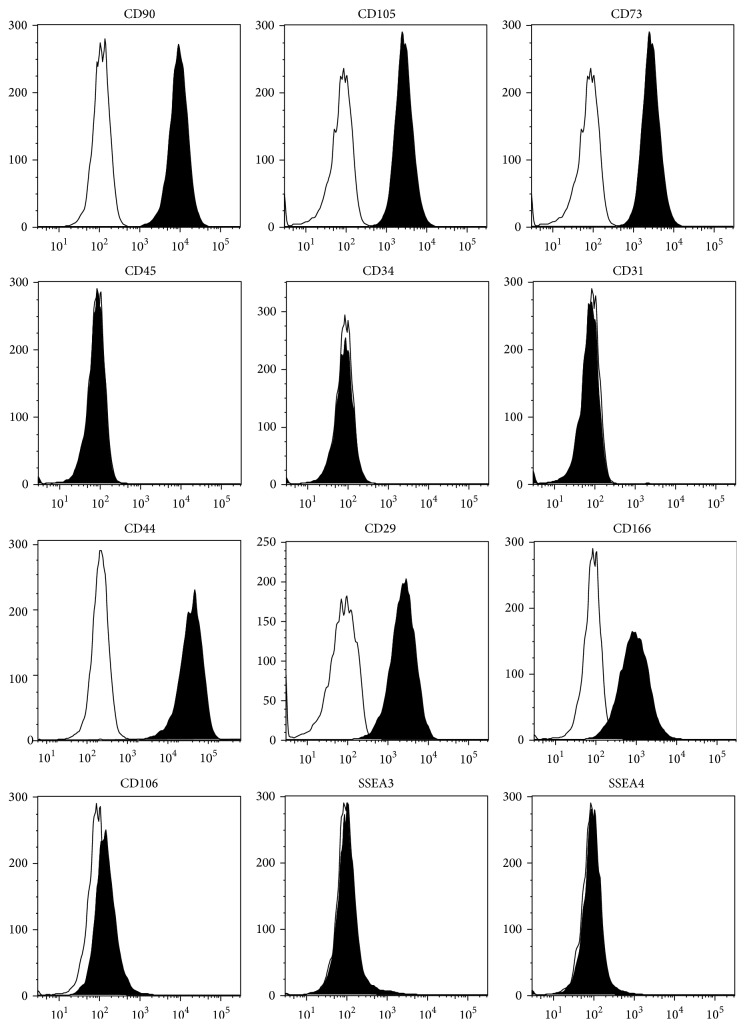
Expression of surface markers in P_3_ hADSCs. The black filled histograms indicate the positive stained cells while the white filled histograms indicate the isotype-matched antibody controls.

Isolated hADSCs from human lipoaspirates were expanded to passage 3 and their specific surface antigens were characterized by flow cytometry in order to ensure the purity of the cell population. Flow cytometry analysis showed that the P_3_ hADSCs highly expressed the mesenchymal stem cell related markers of CD90 (97.6 ± 1.25), CD105 (98.5 ± 0.75), CD73 (94.6 ± 2.75), CD44 (98.3 ± 0.55), CD29 (95.4 ± 1.8), and CD166 (83.3 ± 3.25) and lowly expressed the embryonic stem cell related markers of SSEA3 (4.19% ± 0.84) and SSEA4 (7.16% ± 2.05) but did not express CD45 (0.94% ± 0.35), CD34 (0.63% ± 0.13), or CD31 (0.435% ± 0.08), which indicated that the hematopoietic and endothelial lineages were excluded after culture to P_3_. Additionally, CD106 (0.46% ± 0.18) which was extensively expressed in hBMSCs was weakly positive in hADSCs in agreement with other literatures [20], confirming the specific origin of ADSC from adipose tissue distinct from bone marrow (Figure 1).

### 3.2. Morphology and Biomechanical Property of PGA/PLA Mesh

**Figure 2:**
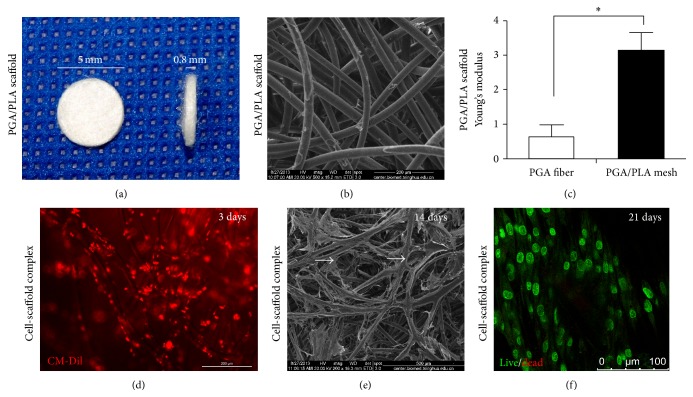
Characterization of PGA/PLA mesh and hADSCs-PGA/PLA complex. Cylinder-shaped PGA/PLA mesh (a). SEM examination of PGA/PLA polymer (scale bars: 200 *μ*m, (b)). Mechanical property of PGA fiber alone and PGA/PLA complex (c). CM-Dil labeled (stained red) hADSCs-PGA/PLA complex 3 days after seeding (scale bars: 200 *μ*m, (d)). SEM examination of hADSCs-PGA/PLA complex with an initial cell seeding density of 10 × 10^6^/mL cultured 14 days after seeding (scale bars: 500 *μ*m, (e)). Live (stained green) and dead staining (stained red) of hADSCs-PGA/PLA complex 21 days after seeding visualized by laser confocal microscopy (scale bars: 100 *μ*m, (f)).

PGA/PLA scaffold was fabricated into a cylinder shape of 5 mm in diameter and 0.8 mm in thickness (Figure 2(a)). SEM showed that the PGA fibers were 30.62 ± 2.8 *μ*m in diameter, and the PLA adhering between polymer fibers assembled the PGA into a mesh with various sizes of pores (~200 *μ*m, Figure 2(b)). PGA coated with PLA exhibited a higher mechanical property in Young's modulus (3.17 ± 0.54 MPa) compared to PGA fibers alone (0.66 ± 0.31 MPa) (Figure 2(c)).

### 3.3. Cell Adhesion, Distribution, and Viability of hADSCs Seeded on PGA/PLA Mesh

hADSCs colored with red cytomembrane by CM-Dil labeling adhered well and distributed homogenously within the polymer fibers at 3 days after seeding (Figure 2(d)). SEM showed that, after 14 days of induction, hADSCs were found self-organized into tubular structures (Figure 2(e), arrows) along with the pores fabricated by the crossed fibers. Live/dead staining assay demonstrated that the hADSCs cultured on PGA/PLA scaffold were all alive with green colored cytoplasm until 21 days of in vitro induction (Figure 2(f)).

### 3.4. In Vitro Endothelial Differentiation of hADSCs

#### 3.4.1. Quantification of Mature Endothelial Phenotype by Flow Cytometry

**Figure 3:**
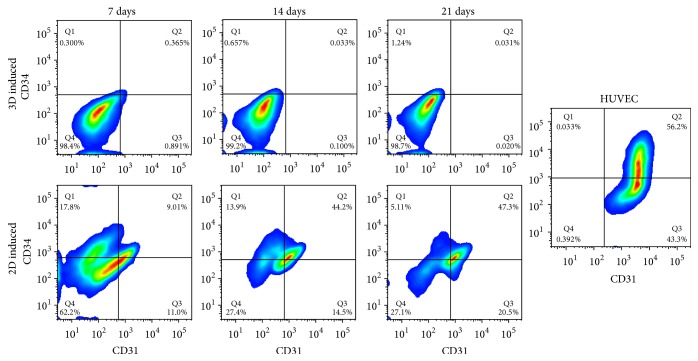
Expressions of CD34 and CD31 in 3D/2D induced hADSCs.

Flow cytometry analysis showed that the percentages of CD34+/CD31+ and CD34−/CD31+ subpopulations in hADSCs cultured on PGA/PLA mesh increased during in vitro induction, whereas the percentages of CD34+/CD31− and CD34−/CD31− subpopulations decreased overtime. Specifically, about 27.5 ± 3.9% in 3D induced hADSCs were detected positive for CD34 and 21.2 ± 3.0% for CD31 after 7 days of induction, and these two markers were both elevated up to 51.9 ± 5.7% and 69.6 ± 8.9% after 21 days, respectively. At the same time, the subpopulation of CD31+/CD34− indicated that the fully endothelial differentiated cells reached a positive ratio of 21.4 ± 3.7%, which was equal to about one-half of that in HUVECs (45.2 ± 1.7%). However, no positive signs for CD34 and CD31 were detected in the 2D induced hADSCs at 7, 14, and 21 days after endothelial induction (Figure 3).

#### 3.4.2. Identification of Endothelial Phenotype by Cytochemical Stainings

**Figure 4:**
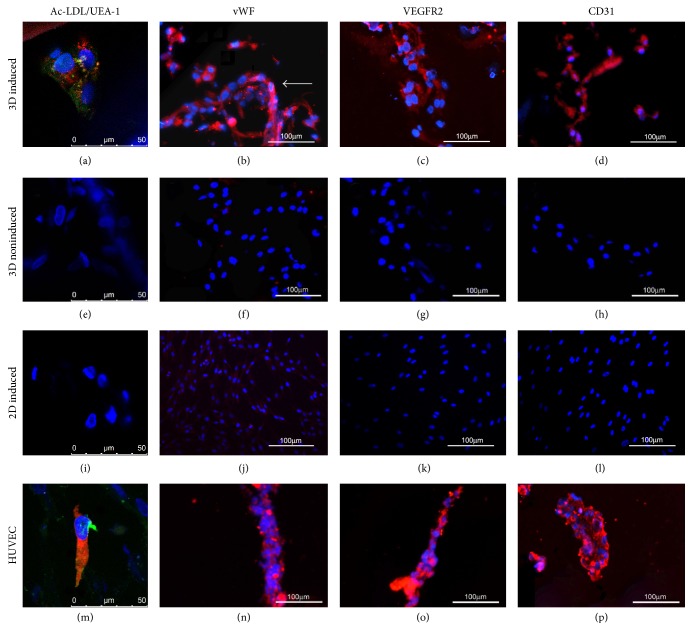
Endothelial phenotype in 2D/3D cultured hADSCs after 21 days. Uptake of Ac-LDL/UEA-1 in the 3D induced hADSCs (a), 3D noninduced hADSCs (e), 2D induced hADSCs (i), and HUEVC (m) at 21 days in vitro examined by laser confocal microscopy (scale bars: 50 *μ*m). Immunofluorescence stainings of endothelial markers of vWF ((b), (f), (j), and (n)), VEGFR2 ((c), (g), (k), and (o)), and CD31 ((d), (h), (l), and (p)) in the 3D induced hADSCs, 3D noninduced hADSCs, 2D induced hADSCs, and HUVEC (scale bars: 100 *μ*m).

The assays of Ac-LDL/UEA-1 uptake and the immunofluorescence of vWF, VEGFR2, and CD31 were performed to analyze the cell differentiation towards the endothelial lineage. Results showed that after 21 days of endothelial induction, the 3D induced hADSCs exhibited double positive stainings in red and green colors, indicating the uptake of Ac-LDL and UEA-1, respectively (Figure 4(a)). Also, hADSCs undergoing endothelial differentiation were found self-organized into a tube-like structure (Figure 4(b), arrow) and stained positive for vWF, VEGFR2, and CD31 (Figures 4(b) to 4(d)). However, except for the expression of vWF, the stainings for VEGFR2 and CD31 were both negative in the 2D induced hADSCs (Figures 4(e) to 4(h)), which was consistent with the result of the flow cytometry analysis. As controls, the hADSCs cultured in 3D conditions without induction failed to gain an endothelial phenotype (Figures 4(i) to 4(l)) while HUVEC showed positive results in all those endothelial markers examinations (Figures 4(m) to 4(p)).

#### 3.4.3. mRNA Expression of Endothelial Markers by Real-Time PCR

**Figure 5:**
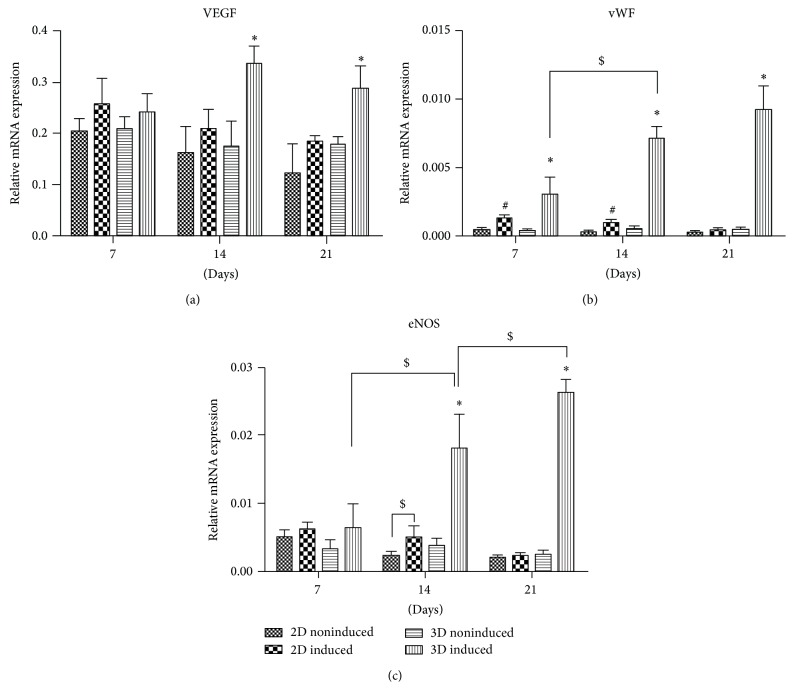
mRNA expression of endothelial related genes. Gene expression of VEGF, vWF, and eNOS in the 3D induced, 3D noninduced, 2D induced, and 2D noninduced hADSCs at 7, 14, and 21 days in vitro. ^∗, #^ *P* < 0.05 means being statistically different from the other 3 groups at the same time point. ^$^ *P* < 0.05 means being statistically different from each other.

Results showed that, during in vitro culture, the mRNA expressions of* VEGF*,* vWF*, and* eNOS* were comparable and stayed at low levels in the noninduced groups under either 2D or 3D condition (Figures 5(a) to 5(c)). However, a weak but significant elevation of* vWF* expression was detected in the 2D induced group after 7 and 14 days of induction compared with the 2D and 3D noninduced groups (Figure 5(b)), as well as a significant increase in the eNOS expression at 14 days compared to the 2D noninduced group (Figure 5(c)). Notably, the expressions of* VEGF*,* vWF*, and* eNOS* in the 3D induced group were all markedly upregulated and significantly different from the other groups at 14 days of induction, indicating that 14 days might be a key time point for hADSCs to obtain the essential endothelial phenotype when cultured on PGA/PLA mesh (Figures 5(a) to 5(c)).

#### 3.4.4. Immunofluorescence Staining and mRNA Expression of *α*-SMA

**Figure 6:**
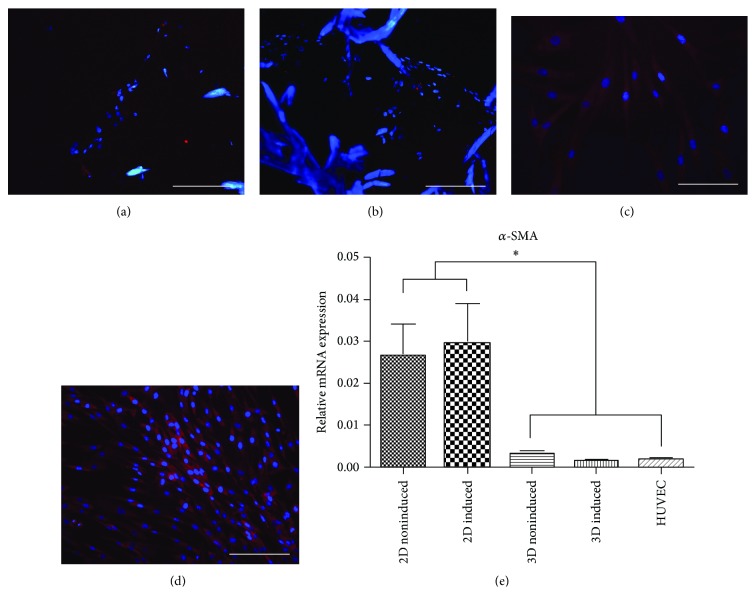
*α*-SMA expression in 2D/3D cultured hADSCs. Immunofluorescence staining of *α*-SMA in 3D noninduced (a), 3D induced (b), 2D noninduced (c), and 2D induced (d) groups after 7 days in vitro. mRNA expression of *α*-SMA in these 4 groups and HUVEC after 7 days in vitro. ^*^ *P* < 0.05 (scale bars: 100 *μ*m).

Results showed that, after 7 days, a stronger positive staining for *α*-SMA was detected in 2D induced hADSC compared to 2D noninduced group, and this expression was vanished in both of the 3D induced and noninduced groups (Figures 6(a) to 6(d)). Gene expression analysis also confirmed the same result in these 4 groups, although no significant difference was found between the 2D cultured groups. Importantly, the expressions for *α*-*SMA* in 3D cultured groups were both significantly different from the 2D cultured groups, staying at very low levels as in HUVEC (Figure 6(e)).

## 4. Discussion

Lack of vascularization is a common obstacle for the in vivo survival of large volume or oxygen consumed engineered tissues. Exploring the proangiogenic function within the construct is a major target, and one of the strategies is the stem cell-based therapy to develop new endothelial cells. As 3D scaffold mediated tissue repair has the advantages of better efficacy in cell migration, differentiation, and matrix deposition, integration of ADSCs in a 3D scaffold might create a construct that features a proangiogenic activity. This study first demonstrated the growth and endothelial differentiation of hADSCs on the PGA/PLA mesh and also pointed out the synergistic effect of growth factors and PGA/PLA 3D culture on the acquisition of mature characteristic endothelial phenotype.

The central roles of 3D scaffold in cell differentiation and tissue regeneration have been highlighted in the literatures. Studies have shown that the isolated cells could hardly organize themselves spontaneously to form complex tissue structures unless the presence of a 3D matrix that guides and stimulates their activities [21]. Compared to matrigel, a matrix commonly used for endothelial differentiation but lacking the porous qualities, 3D polymer mesh provides a better spatial guide to certain cell behaviors, especially like endothelial differentiation which is apt to organize into a tubular structure through cell migration. It was reported that the endothelial differentiation of ADSCs would be enhanced if seeding the cells on the scaffolds modified with nanostructures [22] or built in a specific 3D configuration [23]. Other studies showed MSCs responding to the endothelial differentiation signals also depended on the microstructure [24] and the elasticity of polymer [25]. These findings further implied the high potentials in using PGA/PLA polymer complex as scaffolds for endothelial differentiation, because the composition of PGA and PLA can be easily adjusted and optimized so as to create a structural framework with designated pore size and stiffness.

Liu et al. [26] have reported the influences of PLA contents on cell distribution and ECM production. They pointed out that PGA with different contents (0%, 10%, 20%, and 30%) of PLA showed different pore structures. However, obvious decreases in cell number and ECM deposition were observed in 30% PLA group due to the hydrophobicity of PLA. Therefore, in our experiment we used this experience of PGA coated with 20% of PLA being as a suitable scaffold to ensure not only the shape fixation but also the survival and adhesion of hADSCs. Our results primarily showed that a polymerization of PGA and 20% (w/w) of PLA leaded to a steady framework with a pore diameter less or around 200 *μ*m which enabled hADSCs to form close relationships with their adjacent cells and develop into a tubular-forming tendency. With endothelial induction, hADSC cultured on the porous PGA/PLA mesh was able to gain an endothelial phenotype, demonstrated by the positive expressions of vWF, VEGFR2, CD31, and eNOS at either the cellular or the mRNA levels. Nonetheless, 3D culture without induction still failed to develop any of the endothelial characteristics. This indicated that growth factors were still required for the endothelial differentiation of hADSCs.

The EGM-2 MV culture medium has been often presented as a suitable cell culture environment to trigger the endothelial differentiation of embryonic stem cells and MSCs or the maturation of EPCs. Others have used VEGF in concentrations that ranged from 10 to 50 ng/mL to induce endothelial differentiation [13, 27, 28]. Considering that EGM-2 MV contains a concentration of VEGF lower than 5 ng/mL [29], we added an extra dose of VEGF with 20 ng/mL. However, our results showed that hADSCs cultured on 2D plastic with the above inducing medium were also disabled for endothelial differentiation, as most of the endothelial related markers were lacking after 21 days induction. This result was similar to another study demonstrating that a coseeding of ADSC and b.END-3 onto plastic 2D polystyrene culture plates rather than in matrigel or on a 3D membrane failed to produce any cellular organization reminiscent of a tubulogenic or microvillus network [23]. Except for the 3D induced group, the only positive sign was the inhomogeneously distributed staining of vWF in 2D induced hADSCs concomitant with the slight elevation of* vWF* mRNA expression, which was consistent with Colazzo et al.'s study [12] that the expression of* vWF* was the only gene upregulated compared to* eNOS*,* FLK1*,* FLT-1*, and* CD31* in ADSCs after14 days of 2D induction with 50 ng/mL VEGF. This indicated vWF alone was not specific to symbolize MSC endothelial diferentiation. Besides, we also found that the *α*-SMA staining seemed stronger in 2D induced hADSCs compared to 2D noninduced group. Importantly, it was markedly decreased in 3D cultured groups and stayed at the similar expression level as in HUVEC. van den Akker et al. [30] speculated that 2D endothelial induced porcine BMSCs could be diferentiated toward vascular smooth muscle cells (SMC) rather than endothelium, demonstrated by the expressions of SMC related markers including *α*-SMA. Therefore, it was suggested that hADSCs cytoskeleton could be rearranged or altered through the growth on a 3D polymer scaffold, and the suppression of *α*-SMA might be important for endothelial differentiation.

Collectively, our study presented a synergistic effect of growth factors and PGA/PLA mesh on the acquisition of mature endothelial markers for hADSCs, demonstrated by the significant upregulation of a comprehensive range of specific endothelial markers. However, the expression of CD31 detected by flow cytometry did not reach that level in the HUVEC, which suggested that the inducing approach was still insufficient for fully endothelial transition. This approach would be improved in further studies by the identification of endothelial lineage-specific subpopulations [29], the modification of PGA/PLA mesh with microstructure or chemicals [22], or the combined application of other cytokines [11] and mechanical stress.

## 5. Conclusions

This study first demonstrated the growth and endothelial differentiation of hADSCs on the PGA/PLA mesh and pointed out the synergistic effect of growth factors and PGA/PLA 3D culture on the acquisition of mature characteristic endothelial phenotype. We believe this study would be the initial step towards the generation of prevascularized tissue engineered constructs which might be of great help to the repair of ischemic tissues or the survival for newly regenerated tissues.

## Figures and Tables

**Table 1:** Primer sequences used for real-time PCR.

Gene name	Gene symbol	Forward primer	Reverse primer
Glyceraldehyde-3-phosphate dehydrogenase	*GAPDH *	5-CTGCCCCTTCTGCTGATGC-3	5-TCCACGATGCCGAAGTTGTC-3
Von Willebrand factor	*vWF *	5-TAGAATCCTTACCAGTGACG-3	5-ACTCACACTCATACCCGTTC-3
Endothelial nitric oxide synthase 3	*eNOS *	5-TCACCGCTACAACATCCTG-3	5-CTCATTCTCCAGGTGCTTC-3
Vascular endothelial growth factor	*VEGF *	5-TTCAAGCCATCCTGTGTGC-3	5-ATCTCTCCTATGTGCTGGC-3
*α*-smooth muscle actin	*α*-*SMA *	5-TCTGTAAGGCCGGCTTTGC-3	5-TGTCCCATTCCCACCATCA-3
